# The Effectiveness and Cost-Effectiveness of Community Diagnostic Centres: A Rapid Review

**DOI:** 10.3389/ijph.2024.1606243

**Published:** 2024-01-23

**Authors:** Alesha Wale, Chukwudi Okolie, Jordan Everitt, Amy Hookway, Hannah Shaw, Kirsty Little, Ruth Lewis, Alison Cooper, Adrian Edwards

**Affiliations:** ^1^ Public Health Wales NHS Trust, Cardiff, United Kingdom; ^2^ North Wales Medical School, Health and Care Research Wales Evidence Centre, PRIME Centre, Wales, Bangor University, Bangor, United Kingdom; ^3^ Division of Population Medicine, Health and Care Research Wales Evidence Centre, PRIME Centre Wales, School of Medicine, Cardiff University, Cardiff, United Kingdom

**Keywords:** community diagnostics, secondary care, waiting times, health policy, patient care, effectiveness, cost-effectiveness, review

## Abstract

**Objectives:** To examine the effectiveness of community diagnostic centres as a potential solution to increasing capacity and reducing pressure on secondary care in the UK.

**Methods:** A comprehensive search for relevant primary studies was conducted in a range of electronic sources in August 2022. Screening and critical appraisal were undertaken by two independent reviewers. There were no geographical restrictions or limits to year of publication. A narrative synthesis approach was used to analyse data and present findings.

**Results:** Twenty primary studies evaluating twelve individual diagnostic centres were included. Most studies were specific to cancer diagnosis and evaluated diagnostic centres located within hospitals. The evidence of effectiveness appeared mixed. There is evidence to suggest diagnostic centres can reduce various waiting times and reduce pressure on secondary care. However, cost-effectiveness may depend on whether the diagnostic centre is running at full capacity. Most included studies used weak methodologies that may be inadequate to infer effectiveness.

**Conclusion:** Further well-designed, quality research is needed to better understand the effectiveness and cost-effectiveness of community diagnostic centres.

## Introduction

Community diagnostic centres aim to provide patients with quicker and more convenient direct access to diagnostic services and reduce pressure on hospitals, but evidence of their effectiveness and cost-effectiveness is lacking [1]. The COVID-19 pandemic directly impacted diagnostic services in the United Kingdom (UK) and globally. This, in addition to the rapid rise in demand for diagnostics that existed prior to the pandemic, has resulted in a significant backlog of patients requiring various diagnostic services and increased waiting times. Recently published data showed that in Wales, the number of patients waiting longer than the target of 8 weeks for diagnostics rose from 10.8% (7,964) in March 2020 to 41.5% (44,489) in August 2022 [2]. An Independent Review of Diagnostic Services for NHS England called for significant reform and investment in diagnostic services, and recommended the establishment of community diagnostic centres to aid in tackling the backlog and delays to diagnostic services [3]. With an emphasis on direct patient access to services from primary care, these centres can be located within hospital settings or within the community.

In England, community diagnostic centres were first launched in 2021 in a range of settings including hospitals, football stadiums, and repurposed retail outlets [1]. At present, over 90 community diagnostic centres have been opened, with plans to open up to 160 centres by 2025 [4]. In Wales, a plan to create a network of community diagnostic centres (referred to as Regional Diagnostic Hubs) was outlined by the Welsh Government in April 2022 [5]. As diagnostic services currently account for over 85% of clinical pathways within NHS England and cost over £6 billion per year [6], community diagnostic centres across a range of diagnostic services may be an effective, efficient, and cost-effective intervention for the UK health sector. These services could ensure timely diagnoses and reduced waiting times in a convenient location, ensuring people receive the treatment they need. Furthermore, community diagnostic centres could help address inequalities by providing accessible diagnostic services to people who may be less likely to engage with the healthcare system [7].

Community diagnostic centres are described within the international literature using a variety of terms and definitions. For the purposes of this review, we use the descriptor “diagnostic centres” to incorporate the range of terms used for these services. Diagnostic centres are defined here as health services aimed at improving population health outcomes by providing quicker and easily accessible diagnostic services in the community, which are accessible to primary care practitioners/services, thereby relieving pressure on secondary care services.

This rapid review aimed to examine evidence of the effectiveness and cost-effectiveness of community diagnostic centres. Preliminary work included a rapid evidence summary to identify existing systematic reviews and a rapid evidence map to highlight gaps in the evidence base [8]. This initial investigation identified a lack of recent systematic reviews and a large body of primary evidence with a broad range of outcomes in relation to diagnostic centres. Stakeholders prioritised outcomes relating to the impact of diagnostic centres on capacity and pressure on secondary care, equity in uptake or access, as well as the economic impact of these centres. This review, therefore, focussed on outcomes that were best able to demonstrate this.

## Methods

Rapid reviews accelerate the process of conducting traditional systematic reviews by abbreviating or omitting various steps to produce evidence in an efficient way [9]. The methodology used for this review was developed and used by the Wales COVID-19 Evidence Centre (WCEC) during the coronavirus pandemic to inform policy decisions in Wales. They follow the methodological recommendations and minimum standards for conducting and reporting rapid reviews, including a structured protocol (not published), systematic search, screening, data extraction, critical appraisal, and evidence synthesis [10]. The structure of the review was based on the Preferred Reporting Items for Systematic Reviews and Meta-Analyses (PRISMA) guidelines for systematic reviews [11] to allow for transparent reporting of the approaches used. Patient and public involvement (PPI) within the rapid review context is challenging, especially with the limited time frame for identifying and recruiting relevant people [12]. As part of the WCEC evidence synthesis work, members of the Public Partnership Group (PPG) provided public involvement in each review. This included participating in the stakeholder meetings, informing the review question and scope, commenting on the protocol, contributing to prioritising and defining the outcomes of interest, addressing ongoing queries from the review team, contributing to the executive summary and implications for practice, writing the lay summary, commenting on the mobilisation plan, and supporting the knowledge mobilisation and impact activities.

### Literature Search

The literature search was developed during the preliminary work [8]. Resources searched included MEDLINE, EMBASE (ProQuest), Trip Medical Database (ProQuest), WHO Global, and Google Scholar. The search strategy used to search MEDLINE is available in the Supplementary File. Search concepts and keywords around diagnostic units, centres, hubs, and clinics combined free text words and descriptors when available. Searches were limited to English language publications due to time constraints. References of secondary sources identified during preliminary work were scanned for relevant primary studies and forward and backward citation tracking was conducted.

### Study Selection Process

Studies included in the preliminary work (*n* = 50) were screened for inclusion in this rapid review using the eligibility criteria in Table 1. Screening was undertaken by five independent reviewers (AW,CO,JE,HS,AH) using the systematic review software Rayyan [13]. Disagreements were resolved by discussion amongst the review team.

**TABLE 1:** Eligibility criteria. United Kingdom, August 2022.

	Inclusion criteria	Exclusion criteria
Participants	Symptomatic patients, all conditions being referred to diagnostic centres via primary care settings	
Settings	Diagnostic centres in any setting	Exclude screening programmes, or where there is treatment undertaken but no diagnostics
Intervention	Diagnostic centres/units/hubs and clinics accepting referrals from primary care (as a minimum)	Diagnostic centres accepting referrals exclusively from other routes
Comparison	Usual care/other diagnostic centres	
Outcomes	All outcomes, with a focus on:	
	- capacity	
	- pressure on secondary care	
	- waiting times	
	- equity of access	
	- and all economic outcomes	
Study design	Any design that contains a comparison that can infer effectiveness and economic evaluations	
Countries	All countries	
Language of publication	Studies published in English	Any study not published in English
Publication date	No date limits set	
Publication type	Published and preprint primary literature	All publication types other than primary literature

### Data Extraction

Data extraction and consistency checking were conducted independently by four reviewers (AW, CO, JE, HS). Information extracted included: reference details; study design; intervention/comparator; aim; data collection methods/dates; outcomes measured; study participants; setting; staffing/facilities; services provided; key findings, and any additional relevant notes (Table 2).

**TABLE 2:** Characteristics of included studies grouped by diagnostic centre. United Kingdom, August 2022.

Diagnostic centre(s) (Country)	Study ID	Design	Aim	Condition	Comparator	Participants	Results
Lymphoma Rapid Diagnosis Clinic (LRDC) at Princess Margaret Cancer Centre, Toronto, Ontario (Canada)	[14]	Quasi-experimental cross sectional post-test only	To investigate if wait times can be reduced for a definitive diagnosis of lymphoma and initiation of treatment by implementing a nurse practitioner–led LRDC in a tertiary care cancer centre	Cancer	LRDC patients vs. historical controls	The study included 126 patients referred to the LRDC (no data on historical controls)	The time from initial assessment to lymphoma diagnosis was 16 days (9–24 days) for the patients assessed in LRDC and 28 days (19–48 days) for historical controls (*p* < 0.001). Median time from initial LRDC assessment to treatment of aggressive lymphomas and HL was 29 days (21–43 days) compared with 48 days (28–78 days) for historical controls (*p* < 0.001). Significantly fewer patients required two or more biopsies to arrive at a diagnosis of lymphoma after institution of the LRDC compared with patients previously diagnosed at UHN (40% v 12%; *p* < 0.001). Lymph node size greater than 3.4 cm and presence of mediastinal or abdominal adenopathy increased the likelihood of a diagnosis of malignancy, whereas younger age, being a non-smoker, and prior rheumatologic condition were associated with a non-malignant diagnosis
Rapid diagnostic unit (RDU) at the Gale and Grahem Wright Prostate Centre, North York General Hospital Toronto, Ontario (Canada)	[15]	Quasi-experimental cross sectional post-test only	To document intervals between wait time milestones from suspicion to the start of definitive therapy for patients referred to and treated with radical radiotherapy treatment (RT) at the Odette Cancer Centre, comparing patients diagnosed in the RDU versus the usual community process	Cancer	A multidisciplinary RDU vs. a community-based referral pattern	The study included 44 RDU patients and 43 community patients (controls)	The overall time from suspicion of prostate cancer to RT, was 138 days (RDU cohort) and 183 days (community cohort) (*p* = 0.046). The time from suspicion cancer to diagnosis in the RDU and community cohorts was 49 days and 67 days, respectively (*p* = 0.29). The time from diagnosis to radiation oncology (RO) consult for patients in the RDU and community cohorts was 27 days and 49 days, respectively (*p* = 0.0019). The time from RO consult to start of therapy (RT) for patients in the RDU and community cohorts was 46 days and 37 days, respectively (*p* = 0.52). There were statistically significant differences between the two cohorts, favouring the RDU cohort, for other key wait time intervals. This included suspicion to decision to treat (*p* = 0.012), urologist visit to diagnosis (*p* = 0.0094), diagnosis to decision to treat (*p* = 0.018), and diagnosis to treatment (*p* = 0.016)
Rapid Access Diagnostic and Support (RADS) at The Women’s Breast Health Center, Ottawa Hospital, Ontario (Canada)	[16]	Quasi-experimental cross sectional post-test only	To pilot a rapid diagnosis and support clinic programme for patients referred to the breast centre with a high probability of breast cancer	Cancer	RADS clinic vs. pre-RADS period	The study included 436 women: 211 RADS patients and 225 historical controls	The RADS clinic significantly improved diagnostic wait times and satisfaction scores for patients with a high probability of diagnosis of breast cancer. The mean wait time from abnormal imaging to biopsy decreased by 4.1 days (from 7.1 to 3 days; 58% reduction, *p* < 0.01), biopsy to pathology verification by 0.6 days (from 3.9 to 3.3 days; 15% reduction, *p* < 0.01), pathology verification to surgical consult by 10.1 days (from 16.1 to 5.9 days; 63% reduction, *p* < 0.01), and operative wait time from initial consultation by 7.5 days (from 31.5 to 24.1 days; 24% reduction, *p* = 0.04)
Rapid Access Breast Clinic (RABC) at Mount St Joseph Hospital, Vancouver (Canada)	[17]	Quasi-experimental cross sectional post-test only	To investigate if the RABC will decrease wait times to diagnosis and minimise duplication of services compared to usual care	Cancer	RABC vs. traditional system (TS) i.e., standard care	The study included 64 RABC patients and 178 TS patients	Patients seen at the RABC had a decreased time to surgical consultation (33 vs. 86 days, *p* < 0.0001) for both malignant (36 vs. 59 days, *p* = 0.0007) and benign diagnoses (31 vs. 95 days, *p* < 0.0001). Seventeen (13%) of the patients referred to the surgeon in the traditional system without a diagnosis were eventually diagnosed with a malignancy and waited a mean of 84 days for initial surgical assessment. Of the patients seen at the RABC, 5% required investigation at more than one institution compared to 39% patients seen in usual care (*p* < 0.0001). Cancer patients had a shorted time from presentation to surgery in the RABC (64 vs. 92 days, *p* = 0.009)
Quick Diagnosis Unit (QDU), Hospital Clínic, Barcelona (Spain)	[18]	Quasi-experimental cross sectional post-test only	To investigate the effectiveness and associated costs of a hospital-based ambulatory QDU versus inpatient setting for the diagnosis of pancreatic adenocarcinoma	Cancer	QDU vs. hospitalised patients (inpatient ward at same hospital)	The study included 1,004 (508 QDU patients and 496 inpatients)	Admitted patients were more likely than QDU patients to have weight loss, asthenia, anorexia, abdominal pain, jaundice, and palpable hepatomegaly. Time to admission was significantly shorter than time to the first QDU visit (0.7 [0.2] vs. 1.2 [0.3)] days; *p* < 0.001) and there were no differences between admission time to diagnosis and the QDU time to diagnosis (4.1 [0.8] vs. 4.3 [0.6] days; *p* = 0.163). Considering that the mean admission time to diagnosis of inpatients was 4.1 (1.4) days and that the mean number of visits of QDU patients during the QDU time to diagnosis was 1.02 (0.3), the total cost per hospitalised patient was €634.36 (80.56). With 46.4% being attributable to personnel salaries and 44.2% to diagnostic tests, and the total cost per QDU patient was €347.76 (48.69), with 66.7% being attributable to diagnostic tests, 18.2% to ambulatory visits, and 13.7% to salaries. According to the analysis, the total saving with QDU was €286.6 per patient
As above	[19]	Quasi-experimental cross sectional post-test only	To analyse the main causes of fever as a key or isolated symptom of disease in a cohort of patients evaluated in the QDU of a tertiary university hospital, to examine the advantages and disadvantages of this unit for the diagnosis of fever and to compare the results with a cohort of hospitalised patients	Fever of uncertain nature	QDU vs. hospitalised patients (internal medicine department)	The study included 176 QDU patients and 168 inpatients (controls)	Time to diagnosis of QDU patients was longer than length-of-stay of controls ((25.82 ± 26.14 vs. 12.89 ± 11.33 days, *p* < 0.001). The mean number of visits required to reach a diagnosis in QDU patients was 2.66 ± 1.25 (range: 1–10 visits). 56% patients required one or two visits, while 44% required three or more visits. Patients who required <3 visits had a higher prevalence of infectious diseases, while those who required ≥3 visits had a higher prevalence of inflammatory diseases
							Mean total costs per QDU patient was €644.59 ± 120.18, while it was €4,404.64 ± 815.32 per hospitalised patient. Mean cost per QDU visit was €63.50, and mean cost per day in hospital was €117.00. Direct and Indirect costs varied but were generally less in QDU patients. The mean saving of €3,760 for each QDU patient mostly reflects the differences in staffing and working hours and in number of investigations
As above	[20]	Quasi-experimental cross sectional post-test only	To determine whether quick diagnosis units (QDUs) can safely and efficiently avoid emergency department (ED) visits and hospitalisations	Multiple	QDU vs. hospitalised (internal medicine department)	The study included 4,179 QDU patients and 3.030 hospitalised patients (controls)	Assessment of hospitalised patients concluded hospitalisation might have been avoided in between 84% and 91% of hospitalised patients
							Waiting times to first QDU visit from ED ranged from 0 to 3.5 days (mean 1.9 days) and from PHC ranged from 1 to 7 days (mean 3.6 days). Wait times for admission in hospitalised patients ranged from 0 to 2 days (mean 1.3 days) in those referred from ED and between 3 and 7 days (mean 4.9 days) in those referred from a primary healthcare centre
							The mean cost per process was €3,241.11 (SD 915) in hospitalised patients and €726.47 (617) in QDU patients. Mean cost of hospitalisation per day was €369.99 and mean cost per QDU visit was €232.1
As above	[21]	Quasi-experimental cross sectional post-test only	To describe the functioning of a QDU in a Spanish public university hospital after evaluating 2000 consecutive patients. Authors intended to ascertain the utility and cost of the model compared to conventional hospitalisation and the degree of patient satisfaction	Multiple	QDU vs. hospitalised (admitted to the internal medicine department)	The study included 2,000 consecutive QDU patients and 1,454 control patients	Mean time to diagnosis was 9.4 days (1.78). The authors estimated that 820 (41%) patients would have been candidates for conventional hospitalisation before the QDU was created. Considering mean length-of-stay of the internal medicine department during 2009 for patients admitted for a diagnostic workup was 10.3 days, authors estimated that 12.5 beds per day during a year were freed up (i.e., 4,563 bed-days saved in a year). On the other hand, 4.5% patients required immediate or early hospitalisation due to their bad health status, which impeded further QDU diagnosis. In hospitalised patients, the total mean cost per day of hospital stay was €363.35, and the mean cost per process was €3,153.87 (910). In contrast, the mean cost per process in the QDU was €702.33 (610), and mean cost per QDU visit was €225.83. Mean direct and indirect costs were all less in the QDU visits. Overall satisfaction with QDU care was high in 93% of cases; repeated travel to the hospital was not a major difficulty; and if further diagnostic tests were required, 84% of patients would prefer the QDU care model to hospitalisation
As above	[22]	Quasi-experimental cross sectional post-test only	To compare the diagnostic value and cost of a QDU and conventional hospitalisation in assessing patients with suspected and confirmed cancer in a Spanish tertiary public hospital	Cancer	QDU vs. hospitalisation (internal medicine department)	The study included 169 QDU patients, and 53 hospitalised patients (control)	Time to diagnosis among QDU patients compared to length of stay was not significantly longer 14.4 ± 11.3 days vs. 10.6 ± 9.2 days respectively; *p* > 0.05). The waiting time for the first QDU visit was 2–8 days in PHC referrals and 0–4 days in ED referrals. Waiting times for hospitalised patients were 0–2 days in ED referrals, 2–3 days in referrals from other departments, and 3–8 days in primary healthcare referrals. The mean cost per day of hospital stay was €382.96 in hospitalised patients. The mean cost per visit in QDU patients was €253.94. The mean cost per process was €4,059.37 8,987 in hospitalised patients and €601.84 8,502 in QDU patients
As above	[23]	Quasi-experimental cross sectional post-test only	To investigate the utility and cost of a QDU for the evaluation of patients with severe anaemia (haemoglobin b8 g/l) compared with conventional hospitalisation in a tertiary public hospital in Spain	Severe anaemia	QDU vs. hospitalised patients (internal medicine department)	The study included 282 consecutive QDU patients and 252 consecutive hospitalised patients (controls)	The mean time to diagnosis in QDU patients was 7.82 days (1.36), which was not significantly different from the mean stay of 8.87 (4.45) days of hospitalised patients. Mean haemoglobin and haematocrit were 76.11 (21.8) and 25.03 (6.52), respectively, in QDU patients and 74.61 (21.1) and 24.11 (6.43), respectively, in hospitalised patients. The differences were not statistically significant. Mean length of hospital stay was 8.87 days among hospitalised patients. Total mean cost per day of hospital stay was €2,060.63 in hospitalised patients and €90.04 euros per QDU visit. The mean cost per process was €18,278.01 in hospitalised patients. In contrast, the mean cost per process in the QDU was €2,920.62. There was 92% compliance with the survey of patient opinion. The results highlighted three main aspects: overall satisfaction with QDU care was high in 93% of cases; repeated travel to the hospital was not a major difficulty, and if further diagnostic tests were required, 85% of patients would prefer QDU care to conventional hospital admission
As above	[24]	Quasi-experimental cross sectional post-test only	To describe the functioning of a QDU in a Spanish public university hospital and to ascertain the costs of the QDU model compared to conventional hospitalisation and the degree of satisfaction of QDU patients	Multiple	QDU vs. hospitalised patients (internal medicine department)	The study included 1,000 consecutive patients evaluated in the QDU during time frame	Waiting times for a first QDU visit ranged from 2 to 8 days (mean: 3.9 days) in patients referred from primary healthcare centres and from 0 to 4 days (mean: 2.1 days) in patients referred from the ED of the hospital. Considering that the mean length of stay in the internal medicine department (50 beds) in 2009 for patients admitted for a diagnostic workup was 10.3 days, authors estimated that 12.5 beds/day were made available over the course of a year (i.e., 4,563 bed-days were saved in a year). However, 45 of 1,000 patients (4.5%) required immediate or early hospitalisation due to their bad health status, which impeded further QDU diagnosis. In hospitalised patients, the total mean cost per day of the hospital stay was €356.59 and the mean cost per process was €3,416.13. In contrast, the mean cost per process in the QDU was €735.65
Quick Diagnostic Unit (QDU) at Bellvitge Hospital, Barcelona (Spain)	[25]	Economic evaluation	To evaluate the costs of QDU vs. conventional hospitalisation for the diagnosis of cancer and anaemia using a cost-minimization analysis on the proven assumption that health outcomes of both approaches were equivalent	Multiple	QDU vs. hospitalised patients (internal medicine department)	The study included 195 QDU patients and 237 control patients	The average time to diagnosis at the QDU was 11.1 days. Length of stay of comparable inpatients was 10.3 (+9.1) days. Mean cost saving per patient with a diagnosis of anaemia was €4,422.91 (overall saving €415,753.54), €4,481.41 per patient with a diagnosis of lymphoma (overall saving €282,328.83), and €4,464.13 per patient with a diagnosis of lung cancer (overall saving €169,636.94). Taking into account the mean length of stay and the mean cost per hospital stay, the mean cost saving per patient was €2,956.41. Highest savings for the three groups were related to fixed direct costs of hospital stays (66% of total savings). Savings related to fixed non-direct costs of structural and general functioning were 33% of total savings. Savings related to variable direct costs of investigations were 1% of total savings. Overall savings from hospitalization of all patients were €867,719.31
Quick Diagnosis Unit (QDU), Hospital Plató and Quick Diagnosis Unit (QDU), Hospital Clínic, Barcelona (Spain)	[26]	Economic evaluation	To compare by micro-costing the costs incurred by quick diagnosis units of tertiary and second-level hospitals	Multiple	QDU of tertiary unit (Hospital Clínic) vs. QDU of secondary unit (Hospital Plató)	The study included 407 QDU patients at the Secondary Unit and 407 QDU patients at the Tertiary Unit	The total number of visits in the Tertiary Unit was significantly higher than that in the secondary centre unit (3.098 vs. 2.123, respectively; *p* = 0.0064) the mean time to diagnosis was significantly shorter in the former (8 vs. 12, respectively; *p* < 0.0001). The mean total cost per patient of the Tertiary Unit was €577.50 varying from a minimum of €353.2 to a maximum of €975.8 per patient and year, while the mean cost of the Secondary Unit was €394.70 per patient, ranging from a minimum of €289.6 to a maximum of €539.1 per patient and year. The mean cost per visit of both units was similar. Indirect costs of the Tertiary Unit were significantly higher than those of the secondary centre unit (€49.93 vs. €12.42, respectively; *p* = 0.0018). In both units, direct costs accounted for the largest proportion of cost per patient without significant differences (79.13% in the Tertiary vs. 81.15% in the Secondary Unit; *p* = 0.3327). However, the contribution of indirect costs was significantly greater for the unit of the tertiary centre (8.595% vs. 3.284%, respectively; *p* < 0.0001). Personnel and indirect costs including their percent contribution to overall costs accounted for the main differences
As above	[27]	Quasi-experimental cross sectional post-test only	To comparatively describe the diagnostic performance of the QDU of an urban district hospital (QDU1) and the QDU of its reference general hospital (QDU2)	Multiple	QDU1(Hospital Plató) vs. QDU2 (Hospital Clínic)	The study included 336 patients referred to QDU1 and 530 patients referred to QDU2	The time to first visit was longer in QDU1 than in QDU2 patients (5 vs. 3 days; *p* = 0.008) and the median number of visits was lower in QDU1 patients (2 vs. 2.5, respectively; *p* = 0.003). The QDU2 patients underwent significantly more ultrasonographies, endoscopies, and cytology/biopsy studies than the QDU1 patients. Furthermore, significant differences were observed in the waiting times to CT scan and cytology/biopsy studies, which were longer in QDU2 patients, and in the waiting times to ultrasonography, endoscopy, scintigraphy, and body FDG-PET, which were longer in QDU1 patients. While QDU1 patients were more likely than QDU2 to require ≤2 visits to achieve a diagnosis (73% vs. 57%; *p* < 0.001). the median time-to-diagnosis was longer in the former (12 vs. 8 days, respectively; *p* < 0.001)
Quick Diagnosis Unit (QDU), Hospital Clínic, Barcelona and a Quick Diagnostic Unit (QDU) at Bellvitge Hospital, Barcelona (Spain)	[28]	Quasi-experimental cross sectional post-test only	To investigate the time to diagnosis of a hospital-based outpatient or inpatient setting in four major subtypes of lymphomas and the costs incurred by both clinical settings in the diagnostic process. A further goal was to investigate the frequency, clinical, and prognostic features of each lymphoma subtype according to an outpatient or inpatient diagnosis	Cancer	QDU1 (Hospital Clínic) patients vs. inpatients at Hospital Clínic vs. QDU2 (Bellvitge Hospital) patients	The study included 688 patients from QDU 1, 589 patients from QDU 2, and 535 inpatients (controls)	Inpatients waited less than 24 h to be admitted, whereas time to first visit in outpatients was significantly longer (0.6 vs. 1.7 days; *p* < 0.001). The admission time for diagnosis of inpatients was significantly shorter than the QDU time for diagnosis of outpatients (12.3 vs. 16.2 days; *p* < 0.001). The mean time to biopsy was substantially longer in outpatients (7.4 days) than in inpatients (3.5 days) (*p* < 0.001). The mean admission time for diagnosis of inpatients was 12.3 (3.3) days and that the mean number of visits of outpatients (corresponding to the mean QDU time for diagnosis) was 3.26 (1.2) days. The total cost per hospitalised patient was €4,039.56 (513.02), with 69.5% being attributable to personnel salaries and 25.4% to diagnostic tests. The total cost per outpatient was €1,408.48 (197.32), with 50.6% being attributable to diagnostic tests, 29.5% to outpatient visits, and 18.6% to personnel salaries. According to the analysis, the total saving from hospitalisation was €2,631.08 per patient
One stop clinic at the Breast Care Centre, Bristol (UK)	[29]	Randomised controlled trial	To compare the impact on patients of a one-stop clinic with conventional clinic arrangements involving a minimum of two separate clinic appointments and a delay of several days or weeks before test results are provided	Cancer	One-stop breast lump clinic (providing same-day diagnosis) vs. two-stop clinic (conventional system involving two appointments)	The study included 791 participants (One-stop clinic = 416, Two-stop clinic = 375)	Six days after first clinic attendance the one-stop group showed significantly lower levels of anxiety (*p* < 0.05). However, the sub-group who had breast cancer had become more distressed in both groups, more so in the one-stop group. A benign diagnosis in the one-stop group was associated with fewer symptoms of anxiety (t = −5.47; d.f. = 489; *p* < 0.001), depression (t = −2.68; degrees of freedom (d.f). = 489; *p* < 0.01), improvements on VAS measures of worry about the breast problem (t = 6.08; d.f. = 481; *p* < 0.001), concern about future health (t = 3.13; d.f. = 474; *p* < 0.01), sleeping patterns (t = −5.47; d.f. = 481; *p* < 0.001), concentration (t = −4.69; d.f. = 481; *p* < 0.001), ability to carry on with normal daily activities (t = −3.62; d.f. = 479; *p* < 0.001) and EORTC subscales of quality of life (t = 2.39; d.f. = 471; *p* < 0.05), emotional (t = 4.93; d.f. = 471; *p* < 0.001) and cognitive functioning (t = 2.55; d.f. = 470; *p* < 0.05). Eight weeks later, women receiving a speedier diagnosis of cancer reported higher levels of depression than women given this diagnosis in the two-stop system (*p* < 0.05)
Rapid Diagnostic Clinic (RDC) at St Barts Health NHS Trust, London (UK)	[30]	Quasi-experimental cross sectional post-test only	To critically appraise the efficacy of a RDC with respect to its impact on patients’ timelines and outcomes	Cancer	Rapid Diagnostic Clinic (RDC period) vs. head and neck clinics (pre-RDC period)	The study included 212 patients during the pre-RDC period, and 313 patients during the RDC period	During the pre-RDC period, the mean time taken for patients referred via the 2WW referral system was 11.2 ± 0.6 days (range 1–37 days). The mean time taken for all other target referrals (non-2WW) was 33.5 ± 3.3 days (range 2–145 days). During the RDC period, the mean time taken for patients referred via the 2WW referral system was 9.2 ± 0.4 days (range 1–27 days), and for non-2WW referrals was 23.3 ± 1.9 days (range 1–105 days)
							A comparative data analysis for the timelines from referral to the patients being seen between the pre-RDC and RDC period confirmed a statistically significant reduction in the time from referral to patients first clinic consultation, between the two study periods [referred via the 2WW referral system 11.2 to 9.2 days (*p* = 0.0002); all other referral sources 33.5 to 23.3 days (*p* = 0.0015)]
Demyelinating disease diagnostic clinic (DDC) at University College London (UK)	[31]	Quasi-experimental cross sectional post-test only	To compare a newly established diagnostic clinic with two existing clinical settings in the management of the diagnostic phase of MS.	Multiple Sclerosis	DDC vs. general neurology clinic (GNC) vs. inpatient investigation unit (IIU)	The study included 50 patients (DDC = 20, GNC = 10, IUU = 20)	The time between referral and first appointment favoured the DDC with a mean time of 5.9 weeks, compared to 7.7 weeks for the GNC and 10.0 weeks for the IIU. The mean times between the first appointment and receipt of results were 4.7 weeks (DDC), 18.8 weeks (GNC) and 21.2 weeks (IIU). The price per patient ranged from £395 to £790 (DDC), £95 to £380 (GNC) and £1940 to £2,700 (IIU)
Rapid Diagnostic Centre (RDC) at Neath Port Talbot Hospital, Neath, Wales (UK)	[32]	Economic evaluation	To explore the cost-effectiveness of the RDC compared with standard clinical practice	Cancer	Pilot RDC vs. standard clinical practice	1,000 (simulated patients based on real-life data for intervention and control group)	Mean time to diagnosis was 84.2 days (SD = 65.3) in the control group. This was reduced to 5.9 days (SD = 3.4) in patients who were diagnosed directly at the RDC clinic and to 40.8 days (SD = 30.0) if further investigations following RDC were warranted. Total staff costs per half-day clinic were calculated as £2,640 with CT scan and other test costs amounting to £118.21 per patient
							At between 80% and 100% capacity, the RDC produces more QALYs and is less costly, and thus outperforms standard clinical practice. Below 80% capacity, the RDC is not cost-effective at a £20 000 willingness-to-pay threshold
Community Mobile Diagnostic ultrasound Service throughout the West Midlands (UK)	[33]	Quasi-experimental cross sectional post-test only	To assess the benefits and disadvantages of a radiographer delivered, primary care-based mobile diagnostic ultrasound service by comparing it to an NHS Trust diagnostic ultrasound service	Not specified	Radiographer-led community diagnostic ultrasound service vs. local NHS Trust diagnostic ultrasound service	The study included 200 and 193 adult patients who underwent diagnostic ultrasound in 2001/2002 with the community and NHS Trust services respectively	Mean waiting time for an ultrasound scan appointment was 17.44 (95% CI 15.86–19.02) and 44.53 days (95% CI 38.83–50.23) for the community and NHS Trust services respectively. Location of ultrasound appointment was reported as convenient by 93 (93 per cent) of community service respondents and 78 (95.1 per cent) of hospital service respondents. Time of appointment was reported as convenient by 95 (95 per cent) and 76 (92.7 per cent) of community service and hospital service patients respectively
							Patients were highly satisfied with both services. GPs were markedly less satisfied with the NHS Trust service compared to the community service. Quality of stored ultrasound images and reports were comparable for the services. Cost per abnormality detected was higher for the community service (£107.69 compared to £77.35 for the NHS Trust service, not statistically significant)

### Quality Appraisal

As the designs of included studies were often unclear, the Leatherdale algorithm was used to categorise the studies [34]. A range of study specific Joanna Briggs Institute quality appraisal checklists (randomised controlled trial [35], economic evaluation [36] and quasi-experimental [16]) were used to assess the methodological quality of included studies. Quality assessment and verification of all judgements was undertaken by four reviewers (AW, CO, JE, HS). Disagreements were resolved by discussion amongst the review team. The quality appraisal results can be seen in the Supplementary File.

### Synthesis

This rapid review employed a narrative synthesis approach [24] to describe the impact of diagnostic centres on waiting times, pressures on secondary care, as well as any economic impact, and to explore relationships in the evidence found. The use of meta-analysis to synthesise quantitative findings was considered, however, due to the heterogeneity of included studies, it was not possible to undertake a valid meta-analysis. Stakeholders highlighted the importance of identifying if diagnostic centres could impact waiting times, pressures on secondary care, as well as any economic impact. As such, the outcomes identified were categorised into “impact on waiting times,” “impact on pressure” and “economic outcomes.” Where multiple studies reported outcomes on the same diagnostic centre, only the findings from the most recent study were reported to avoid the risk of double counting.

## Results

**FIGURE 1:**
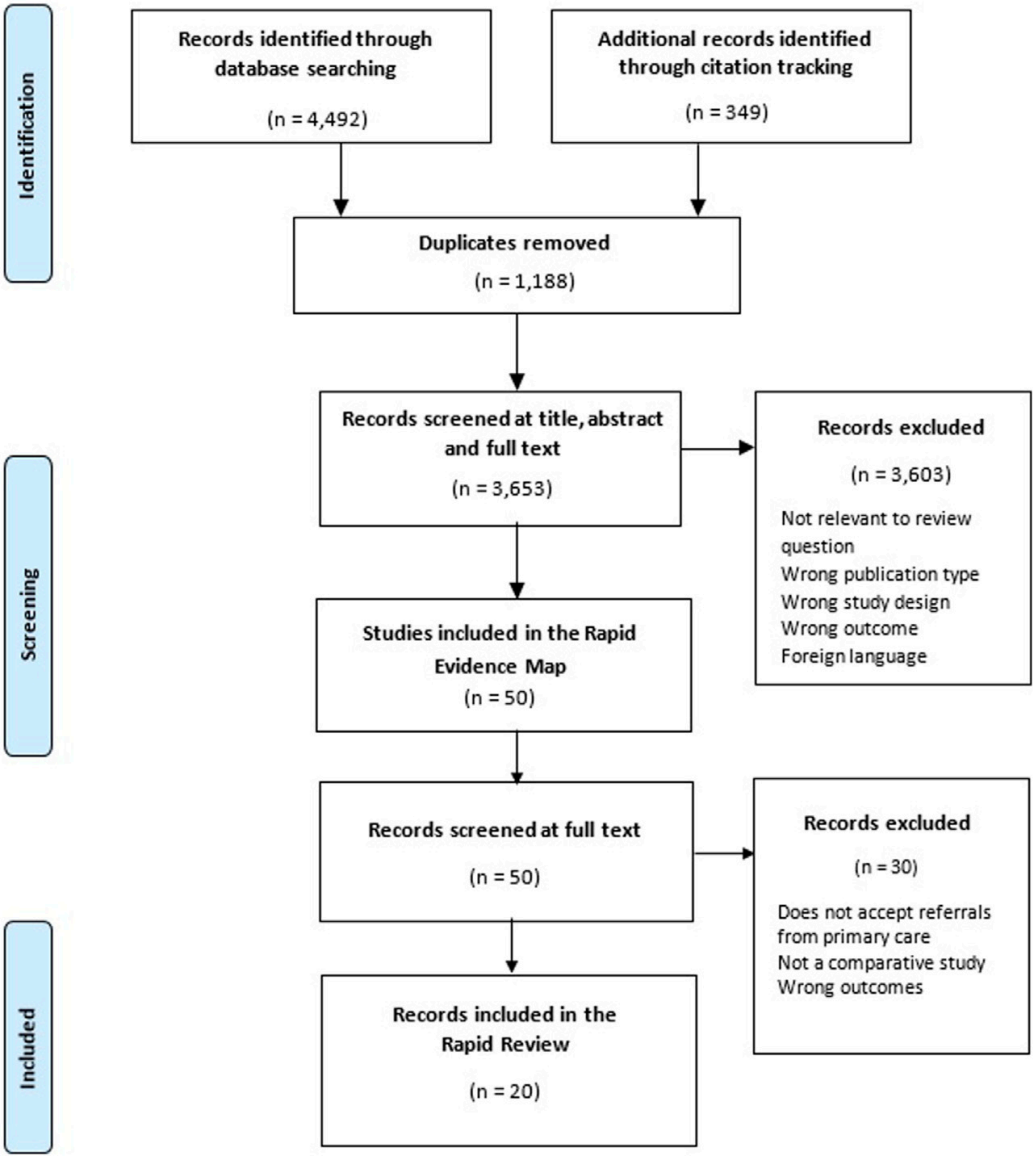
Preferred reporting items for systematic reviews and meta-analyses (PRISMA) diagram for included studies. United Kingdom, August 2022 [11].

A total of 20 studies was included, reporting data from 12 individual diagnostic centres. The study selection process is shown in Figure 1. Table 2 contains the characteristics of included studies grouped by the diagnostic centre they report on. Details about the characteristics of each diagnostic centre can be found in the Supplementary File. Sixteen quasi-experimental studies [14, 15, 17–23, 25–28, 30, 31, 33], three economic evaluations [29, 32, 37] and one randomised controlled trial (RCT) [38] were included. The studies were conducted in Spain (*n* = 11), UK (*n* = 5), and Canada (*n* = 4), and were published between 1998 and 2021. Most studies described diagnostic centres located within hospital settings (*n* = 19) with only one study describing a diagnostic service located within the community setting [15]. Ten studies were specific to cancer diagnoses [14, 19–21, 25, 27, 30, 31, 37, 38], six reported on the diagnosis of several health conditions [18, 22, 23, 29, 32, 33] and three studies covered a single health condition including severe anaemia (*n* = 1) [28], fever of uncertain nature (FUN) (*n* = 1) [17], and multiple sclerosis (MS) (*n* = 1) [26]. One study did not report a health condition of interest [15]. All studies had considerable methodological limitations and the quality of reporting was often poor. A range of performance and economic outcomes was reported and a detailed matrix of the outcomes reported by each included study can be found in the Supplementary File.

### Impact of Diagnostic Centres on Waiting Times

Nineteen studies reported outcomes relevant to waiting times [14, 15, 17–23, 25–33, 37]. These related to different intervals of the diagnostic and treatment pathway. Some time intervals were poorly defined across the studies, to avoid misinterpretation they have been reported separately.

Time to first visit (the interval between primary care referral and first visit to the diagnostic centre) was reported for five diagnostic centres [19, 26, 27, 30, 33]. Findings were generally mixed. A reduction in time to first visit was reported in two studies for diagnostic centre patients compared to historical controls [26, 27], however the difference was only statistically significant in one study [27]. Two studies reported that the time to first visit was statistically significantly longer than the time to hospital admission for inpatients [19, 30]. When comparing two diagnostic centres, statistically significant differences were found in the median time to first visit, which was longer in the diagnostic centre of an urban district hospital than that of the diagnostic centre in a tertiary hospital [33].

Time to examination (the interval between diagnostic centre physician’s order and the examination being performed) was reported for four diagnostic centres [15, 19, 28, 33]. Study findings were generally mixed. Two studies found the mean time to examination at a diagnostic centre was shorter when compared with usual care [15, 28], while another study found a statistically significant difference in the mean time to biopsy, which was longer in the diagnostic centre than in inpatient settings [19]. When comparing two diagnostic centres, statistically significant differences were found in the time to computed tomography (CT) scan and cytology/biopsy tests, which were longer in the diagnostic centre of a tertiary hospital, while time to ultrasonography, endoscopy, scintigraphy, and positron emission tomography were longer in the diagnostic centre of an urban district hospital [33].

Time to diagnosis (the interval between the request of the decisive diagnostic procedure and the cyto/pathological diagnosis) was reported for six diagnostic centres [19, 25, 30–32, 37]. Findings were generally mixed. Three studies found the time to diagnosis to be shorter for diagnostic centre patients compared with usual care [25, 37] or historical controls [31], (two of which reported statistically significant differences [25, 31]). One study found no significant difference in the time to diagnosis between diagnostic centre and hospitalised patients [30] and one study found the time to diagnosis for diagnostic centre patients to be statistically significantly longer than for hospitalised patients [19]. When comparing different diagnostic centres a statistically significant difference was reported in the time to diagnosis, which was longer in the diagnostic centre of an urban district hospital than in the diagnostic centre of a tertiary hospital [32].

One study reported on the wait time from abnormal imaging to biopsy and from biopsy to pathology verification. Statistically significant reductions in the mean wait time for both time intervals for diagnostic centre patients compared with historical controls were identified [21].

Time to surgical consultation was reported for two diagnostic centres [14, 21]. However, these studies used different start points to measure this outcome. One study reported a statistically significant reduction in the time from pathology verification to surgical consultation for diagnostic centre patients compared to historical controls [21]. While the other reported a statistically significant decrease in the time from presentation at the clinic to surgical consultation for both malignant and benign diagnoses when attending a diagnostic centre compared to usual care [14].

The time from cancer suspicion to treatment (from suspicion by the physician or patient to radiotherapy) was reported by one study which found a statistically significant reduction in the time interval for diagnostic centre patients compared to the usual community referral process [25].

Time from consultation to therapy (from consultation with a surgeon or consultant within the diagnostic centre to treatment/surgery) was reported for five diagnostic centres [14, 21, 25, 27, 31], all focussed on the diagnosis of cancer. Two studies reported a reduction in the time from surgical consultation to surgery for diagnostic centre patients compared to historical controls [21] or usual care [14]. However, the reduction was only statistically significant in one of the studies [21]. One study reported a reduction in the time from first consultation at the diagnostic centre to the date of surgery when compared with historical controls, although this was not statistically significant [27]. Two studies reported a reduction in the time from consultation at the diagnostic centre to the start of treatment when compared to historical controls [17] or usual care [25] with one of these studies reporting the reduction to be statistically significant [31].

One study reported the time from diagnosis to radiation oncology consult was shorter for diagnostic centre patients compared to the usual community referral process and that this difference was statistically significant [25]. The same study also reported the time from diagnosis to radiation therapy and found a statistically significant reduction in time for patients attending the diagnostic centre [25].

### Impact of Diagnostic Centres on Capacity and Pressure on Secondary Care

Thirteen studies reported a range of outcomes relevant to the impact of diagnostic centres on capacity and pressure on secondary care [14, 17–20, 22, 23, 26–28, 31–33].

The number of diagnostic centres patients attended to receive a diagnosis was reported by one study, which found that compared to usual care, statistically significantly more diagnostic centre patients were able to receive a diagnosis from one diagnostic centre and were not required to visit other centres for diagnostic tests [14].

The number of visits to a diagnostic centre required to obtain a diagnosis was reported for three diagnostic centres across two studies [26, 32]. Findings were inconsistent. One study reported that diagnostic centre patients required on average two visits before receiving a diagnosis compared to one to four visits, and two to five visits respectively, for the other clinical settings studied [26]. When comparing two diagnostic centres one study found that statistically significantly fewer visits were required to achieve a diagnosis at the diagnostic centre located within an urban district hospital compared to the diagnostic centre within a tertiary hospital [32].

The number of biopsies required to arrive at a definitive diagnosis was reported for one diagnostic centre [31]. Fewer patients required two or more biopsies to arrive at a diagnosis of lymphoma after the introduction of a diagnostic centre compared to usual care, and that this difference was statistically significant [31].

Referral patterns were reported for one diagnostic centre and showed statistically significant differences overtime, with more direct referrals being made to the diagnostic centre from emergency departments and less patients being hospitalised [18]. In addition, 84%–91% of hospitalised patients were found to be suitable to attend the diagnostic centre and could have avoided hospitalisation [18].

Onward referrals were reported for four diagnostic centres across three studies [19, 27, 33]. One study reported that after lymphoma diagnosis, diagnostic centre patients were statistically significantly more likely to be referred to outpatient specialist clinics and less likely to be referred to palliative care [19]. However, the authors acknowledged this was likely related to inpatients generally being older and having more aggressive lymphoma subtypes than diagnostic centre patients [19]. When comparing two diagnostic centres, those diagnosed in the diagnostic centre of the tertiary hospital were more likely to be referred to primary care centres or to the tertiary hospital’s specialised outpatient clinics compared to patients diagnosed in the diagnostic centre in an urban district hospital [33]. One study reported an increase in the number of patients in whom a definitive outcome was reached (discharged or being listed for surgery) from 33% of historical controls to 48% of diagnostic centre patients. Additionally, the number of patients requiring onward referral fell by more than half [27].

### Economic Impact of Diagnostic Centres

Fourteen studies reported economic outcomes for seven diagnostic centres [15, 17–20, 22, 23, 26, 28–30, 32, 37, 38]. Of these, three were economic evaluations: one cost-minimisation analysis [25]; one cost-effectiveness study [37]; and one comparative cost analysis [32]. The other 11 quasi-experimental studies reported more generic cost data. In an attempt to highlight the more robust methodological studies (economic evaluations), these findings are reported first.

### Economic Evaluations

One study used patient-level discrete-event simulation and decision analytic modelling to estimate the cost-effectiveness of a pilot diagnostic centre in its first year of operation compared with standard clinical practice in the UK [37]. During the start-up phase, the diagnostic centre saw a mean number of 2.78 patients per clinic and was more costly and more effective compared to standard clinical practice with an incremental cost-effectiveness ratio of £29,732. However, when run at near or full capacity (80% or higher, seeing a mean number of 4/5 patients/clinic), the diagnostic centre was found to outperform usual care, i.e., being less costly and more effective (incremental cost-effectiveness ratio of −£1,775/−£16,124) [37].

A comparative cost analysis using micro-costing was conducted to compare the costs incurred by two diagnostic centres located within different hospitals in Spain [32]. The mean total cost per patient in the tertiary hospital was €577.50 ± 219.60, compared to €394.70 ± 92.58 in the urban district hospital, although the mean cost per visit to both centres was similar (€182.8 ± 41.47 vs. €184.6 ± 29.41 respectively). The direct and structural costs per patient at the two centres were not significantly different. However, the indirect costs of the tertiary hospital were statistically significantly higher than those of the urban district hospital (€49.93 ± 19.90 vs. €12.42 ± 2.344 respectively). The main driver of the cost differences between the two diagnostic centres was the total number of visits and successive/first visits ratio [32].

A cost-minimisation analysis was conducted to assess the costs of the diagnostic centre approach compared with the costs of conventional hospitalisation in Spain [29]. Three groups of diagnostic centre patients (with a final diagnosis of severe anaemia, lymphoma, and lung cancer) were compared with hospitalised patients with the same diagnoses. The results showed cost savings of care delivered by the diagnostic centre compared with traditional inpatient care. The savings from hospitalisation were related to the direct costs of hospital stays (66% of savings), the non-direct costs of structural and general functioning (33% of savings) and the cost of diagnostic investigations (1% of savings). Overall savings from hospitalisation of all patients was €867,719.31 [29].

### Generic Cost Data Reported in Quasi-Experimental Studies

The mean cost per diagnostic centre visit was reported for three different diagnostic centres [19, 26, 30]. Two studies reported the cost per hospital stay to be cheaper than the cost per visit to a diagnostic centre in Spain [19, 30]. A UK study found that the cost per appointment to the diagnostic centre was more expensive compared to per visit to an outpatient clinic (£395 vs. £95) but both were cheaper compared to inpatients, where the length of stay ranged from one to 5 days, with admission and testing costing £1,750 [26].

The cost of diagnostic tests per patient was reported for three diagnostic centres [15, 19, 30]. The findings suggested diagnostic tests may be cheaper in diagnostic centres situated within hospital grounds. Two studies found the total cost of diagnostic examinations per patient to be statistically significantly cheaper for diagnostic centre patients compared to hospitalised patients in Spain [19, 30]. Whereas in the UK, for patients attending the mobile diagnostic ultrasound service in the community, the cost of diagnostic tests per patient was found to be more expensive compared to the NHS Trust hospital service (£30 vs. £20.62–£27.51; respectively) [15].

The total cost per patient was reported for two diagnostic centres in Spain [19, 30]. For both centres, the total cost per patient was statistically significantly less than the total cost per hospitalised patient [19, 30]. It was shown that in the diagnostic centre 66.7% of the cost was attributable to diagnostic tests, 18.2% to ambulatory visits, and 13.7% to salaries, while the total cost per hospitalised patient included 46.4% being attributable to personnel salaries and 44.2% to diagnostic tests [30]. The average cost per process was reported for one diagnostic centre which showed the average cost to be more expensive for hospitalised patients compared to diagnostic centre patients (€3,241.11 vs. €726.47) [18].

Direct, indirect and structural costs were reported for one diagnostic centre [30]. The mean non-direct costs per patient were found to be statistically significantly less in the diagnostic centre compared to hospitalised patients and mainly corresponded to structural and general functioning costs [30]. Staffing costs were reported for two diagnostic centres, both of which identified a statistically significant reduction in staff costs compared to hospitalisation [19, 30]. Lastly, one study combined and analysed data for two diagnostic centres as a single unit and reported the total cost saving from hospitalisation to be €2,631.08 per patient [19].

## Discussion

### Summary of Key Findings

This review sought to examine the evidence on the effectiveness and cost-effectiveness of diagnostic centres with a particular interest in those set within the community. However, only one diagnostic service located within the community was identified, while the remaining studies covered diagnostic centres located in hospitals. Overall, the impact of diagnostic centres on waiting times appears to be mixed. The evidence suggests that diagnostic centres can reduce various waiting times, including time to surgical consultation and time from consultation to treatment. However, the evidence was mixed for other wait time outcomes including the time to first visit, time to diagnostic examination and time to diagnosis. Reductions in waiting times were reported for a number of additional intervals, although these outcomes were reported by individual studies and as such, firm conclusions cannot be made. Reducing wait times would speed up the diagnostic pathway, and could reduce the backlog of patients waiting, thereby reducing pressure in secondary care. However, although diagnostic centres may reduce diagnostic wait times, the ability to reduce the time to treatment is dependent on the capacity of the system to provide treatment. Evidence relating to the impact of diagnostic centres on capacity and pressure in secondary care in this review appears to be unclear. The evidence suggests that diagnostic centres may reduce the number of visits or the number of biopsies needed to receive a definite diagnosis, increase the number of patients reaching a clear management plan and could reduce the number of patients being referred for hospitalisation over time. However, these findings were reported by individual studies and as such firm conclusions cannot be made.

The evidence from this review suggests that diagnostic centres are cost-saving, and may be a more cost-effective resource than traditional inpatient care. However, it appears that overall cost-effectiveness may be dependent on whether the diagnostic centre is running at full capacity. Factors that could determine the costs incurred by a diagnostic centre include the diagnostic and clinical complexity of the patients, as well as the characteristics of the centre including the number of staff and contribution of staff time. Additionally, there is evidence to suggest that diagnostic centres can reduce staffing costs, costs incurred per patient, and the costs of diagnostic tests.

### Findings in Relation to Previous Research

The paucity of evidence for community-based diagnostic services found in this review is in keeping with a previous literature mapping exercise and focused review [39], which identified a limited evidence base for community diagnostic services, suggesting that internationally, diagnostic centres are not commonly set up within the community. This reflects the current establishment of these sites across England, with only one in five centres opened in a community setting rather than on existing healthcare sites [7]. Whilst siting a diagnostic centre within a hospital is likely to provide greater availability to established and functioning diagnostic equipment and services, it may not be accessible to everyone and could worsen existing health inequalities. The concept of *“distance decay”* is recognised within the literature, with a systematic review of global north countries suggesting that those who live further away from healthcare services may have worsening healthcare outcomes [40].

However, there are potential benefits of diagnostic centres that are not dependent on their location. The literature has suggested that diagnostic centres can reduce waiting times to diagnostic tests and increase patient satisfaction [41]. A previous systematic review also found that diagnostic centres resulted in savings from hospitalisation ($2,336–$3,304) [42] supporting the findings from this review. The majority of included studies in this review compared diagnostic centre patients with a range of comparators including hospitalised patients and historical controls. These comparisons may not be appropriate considering that hospitalised patients are generally more acutely unwell and require more clinical input and longer care than those eligible to attend a diagnostic centre. This is supported by a narrative review that suggested diagnostic centres (specifically quick diagnostic centres) may be a more suitable option instead of hospitalisation for general healthy patients with suspected severe conditions [43].

### Strengths and Limitations of This Rapid Review

Strengths of this rapid review include the use of a comprehensive search strategy incorporating extensive electronic database searches and review of secondary sources identified during preliminary work, quality assessment of included studies using appropriate quality appraisal tools, and the systematic approach to reporting of review findings in compliance with PRISMA guidelines.

As this is a rapid review, the methods used have been less vigorous than that of a traditional systematic review. There is therefore, the possibility that some relevant research could have been missed and there is the potential for publication bias.

### Strengths and Limitations of the Available Evidence

All included studies had clear aims and objectives, and the majority had relatively large sample sizes. However, much of the evidence was derived from quasi-experimental studies with considerable methodological limitations. Key details pertaining to outcome measures or information about diagnostic centres were often lacking or poorly reported. In addition, key statistical parameters, such as confidence intervals, were not reported in some studies, making it difficult to determine the magnitude of effect of some diagnostic centres. The majority of included studies were conducted in Spain, which could limit the generalisability of our findings due to differences in healthcare systems and healthcare provision. Furthermore, many of the studies conducted in Spain reported data from the same diagnostic centres, with similar data collection periods, thereby creating the potential for double counting (see Supplementary File for details about the potential for data overlap). No studies explored equity of access to diagnostic centres and only three economic evaluations were identified, highlighting a need for further research in this area.

The diagnostic centres identified in this review varied in terms of the setting, staffing, and condition being diagnosed, further limiting the generalisability of the results. Each diagnostic centre had an individual aim, with some aiming to speed up access to diagnostic testing and overall diagnosis, and others aiming to support the patient journey by ensuring all tests can be accessed in a single day and providing follow-up support (see Supplementary File).

### Implications for Policy and Practice

In light of the paucity of robust evidence, further well-designed, higher quality research is needed to better understand the effectiveness of community diagnostic centres. Research around diagnostic centres sited outside of hospital locations is particularly needed to investigate the impact on equity of access as well as the optimum location for siting these centres. In addition, further research is required to evaluate the effectiveness of diagnostic centres for conditions other than cancer, and full economic evaluations of these centres are also needed to better understand how diagnostic centres can be efficiently utilised. Policymakers would need to consider the feasibility and practicality of incorporating diagnostic centres into the healthcare sector. This would include the availability of funding for these diagnostic centres, adequate planning (including the siting of these centres), and greater inter-sector co-operation between the NHS and private sector.

### Conclusion

This rapid review has highlighted possible benefits of diagnostic centres, particularly with regards to their impact on waiting times and pressure on secondary care. Although inferences around the effectiveness of community diagnostic centres cannot be made due to the paucity of evidence from diagnostic centres located outside of hospital settings, the information extracted from these studies provide valuable information into the potential benefits of establishing these centres. As diagnostic centres continue to be opened across the UK, comparative impact evaluations should be incorporated into service development plans from the onset, to assess the effectiveness of these diagnostic centres over time.
