# Supplementary material for: The Effectiveness and Cost-Effectiveness of Community Diagnostic Centres: A Rapid Review
Source: Int J Public Health. 2024 Jan 23;69:1606243. doi: 10.3389/ijph.2024.1606243 (PMC10844947; doi:10.3389/ijph.2024.1606243)
Supplement: Supplementary file 1 [file DataSheet1.docx]

## Supplementary File

**1. Search strategy used for Medline**

| **Set#** | **Searched for** | **Results** |
| --- | --- | --- |
| S1 | ((TI,AB("community diagnos* centre*"))) | 1 |
| S2 | ((TI,AB("community diagnos* clinic*"))) | 1 |
| S3 | ((TI,AB("community diagnos* hub*"))) | 1 |
| S4 | ((TI,AB("community diagnos* unit*"))) | 0 |
| S5 | ((TI,AB("rapid diagnos* unit*"))) | 16 |
| S6 | ((TI,AB("rapid diagnos* clinic*"))) | 12 |
| S7 | ((TI,AB("rapid diagnos* centre*"))) | 4 |
| S8 | ((TI,AB("rapid diagnos* hub*"))) | 0 |
| S9 | ((TI,AB("mobile diagnos* hub*"))) | 0 |
| S10 | ((TI,AB("mobile diagnos* clinic*"))) | 0 |
| S11 | ((TI,AB("mobile diagnos* centre*"))) | 0 |
| S12 | ((TI,AB("mobile diagnos* unit*"))) | 8 |
| S13 | ((TI,AB("multidisciplinary diagnos* unit*"))) | 0 |
| S14 | ((TI,AB("multidisciplinary diagnos* hub*"))) | 0 |
| S15 | ((TI,AB("multidisciplinary diagnos* centre*"))) | 4 |
| S16 | ((TI,AB("multidisciplinary diagnos* clinic*"))) | 2 |
| S17 | ((TI,AB("mobile healthcare unit*"))) | 2 |
| S18 | ((TI,AB("accelerate coordinate evaluate"))) | 3 |
| S19 | S18 OR S17 OR S16 OR S15 OR S14 OR S13 OR S12 OR S11 OR S10 OR S9 OR S8 OR S7 OR S6 OR S5 OR S4 OR S3 OR S2 OR S1 | 52 |
| S20 | ((TI,AB("diagnos* centre*"))) | 408 |
| S21 | ((TI,AB("diagnos* clinic"))) | 282 |
| S22 | ((TI,AB("diagnos* clinics"))) | 86 |
| S23 | ((TI,AB("diagnos* hub*"))) | 7 |
| S24 | ((TI,AB("diagnos* unit*"))) | 462 |
| S25 | S24 OR S23 OR S22 OR S21 OR S20 OR S19 | 1247° |
| S26 | (MJMESH.EXACT("Diagnostic Services")) | 1211° |
| S27 | S26 OR S25 | 2436° |

**2. Quality appraisal tables**

Quality appraisal results for economic evaluations

| **Study** | **JBI Appraisal Items – Economic evaluations** | | | | | | | | | | |
| --- | --- | --- | --- | --- | --- | --- | --- | --- | --- | --- | --- |
|  | **Q1** | **Q2** | **Q3** | **Q4** | **Q5** | **Q6** | **Q7** | **Q8** | **Q9** | **Q10** | **Q11** |
| Bosch et al 2021 | Y | Y | Y | U | Y | Y | Y | U | N | Y | N |
| Sanclemente-Ansó et al 2016 | Y | Y | Y | Y | Y | Y | Y | U | N | Y | N |
| Sewell et al 2020 | Y | Y | Y | Y | Y | Y | N | N | Y | Y | N |
| Key: Y=Yes, N=No, U=Unclear, N/A=not applicable | | | | | | | | | | | |

Q1. Is there a well defined question?

Q2. Is there comprehensive description of alternatives?

Q3. Are all important and relevant costs and outcomes for each alternative identified?

Q4. Has clinical effectiveness been established?

Q5. Are costs and outcomes measured accurately?

Q6. Are costs and outcome valued credibly

Q7. Are costs and outcomes adjusted for differential timing?

Q8. Is there an incremental analysis of costs and consequences?

Q9. Were sensitivity analysis conducted to investigate uncertainty in estimates of cost or consequences?

Q10. Do study results include all issues of concern to users?

Q11. Are the results generalizable to the setting of interest in the review?

Quality appraisal results for randomised controlled trials

| **Study** | **JBI Appraisal Items – Randomised Controlled Trial** | | | | | | | | | | | | |
| --- | --- | --- | --- | --- | --- | --- | --- | --- | --- | --- | --- | --- | --- |
|  | **Q1** | **Q2** | **Q3** | **Q4** | **Q5** | **Q6** | **Q7** | **Q8** | **Q9** | **Q10** | **Q11** | **Q12** | **Q13** |
| Harcourt et al 1998 | Y | Y | Y | N | N | N | Y | Y | Y | Y | Y | Y | Y |
| Key: Y=Yes, N=No, U=Unclear, N/A=not applicable | | | | | | | | | | | | | |

Q1. Was true randomization used for assignment of participants to treatment groups?

Q2. Was allocation to treatment groups concealed?

Q3. Were treatment groups similar at the baseline?

Q4. Were participants blind to treatment assignment?

Q5. Were those delivering treatment blind to treatment assignment?

Q6. Were outcomes assessors blind to treatment assignment?

Q7. Were treatment groups treated identically other than the intervention of interest?

Q8. Was follow up complete and if not, were differences between groups in terms of their follow up adequately described and analysed?

Q9. Were participants analysed in the groups to which they were randomised?

Q10. Were outcomes measured in the same way for treatment groups?

Q11. Were outcomes measured in a reliable way?

Q12. Was appropriate statistical analysis used?

Q13. Was the trial design appropriate, and any deviations from the standard RCT design (individual randomization, parallel groups) accounted for in the conduct and analysis of the trial?

Quality appraisal results for quasi-experimental studies

| **Study** | **JBI Appraisal Items – Quasi-experimental studies** | | | | | | | | |
| --- | --- | --- | --- | --- | --- | --- | --- | --- | --- |
|  | **Q1.** | **Q2.** | **Q3.** | **Q4.** | **Q5.** | **Q6.** | **Q7.** | **Q8.** | **Q9.** |
| Arnaout et al 2013 | Y | U | Y | Y | N | N/A | Y | Y | Y |
| Bosch et al 2011 | Y | U | Y | Y | N | N/A | Y | Y | N/A |
| Bosch et al 2012a | Y | N | Y | Y | N | N/A | N | Y | Y |
| Bosch et al 2012b | Y | N | Y | Y | N | N/A | Y | Y | Y |
| Bosch et al 2012c | Y | N | Y | Y | N | N/A | N | Y | Y |
| Bosch, Jordán and López-Soto 2013 | Y | N | Y | Y | N | N/A | N | Y | Y |
| Bosch et al 2018 | Y | N | Y | Y | N | N/A | Y | Y | Y |
| Bosch et al 2020 | Y | N | Y | Y | N | N/A | Y | Y | Y |
| Brito-Zerón et al 2014 | Y | N | Y | Y | N | N/A | N | Y | Y |
| Choudhury et al 2013 | Y | U | Y | Y | N | N/A | Y | Y | Y |
| McKevitt et al 2017 | Y | U | Y | Y | N | N/A | Y | Y | Y |
| Montori-Palacín et al 2017 | Y | N | Y | Y | N | N/A | Y | Y | Y |
| Nixon et al 2019 | Y | U | Y | Y | N | N/A | Y | Y | Y |
| Pallan et al 2005 | Y | U | Y | Y | N | N/A | Y | U | U |
| Porter et al 2003 | Y | N | Y | Y | N | N/A | Y | U | N/A |
| Sethukavalan et al 2013 | Y | Y | Y | Y | N | N/A | Y | Y | Y |
| Key: Y=Yes, N=No, U=Unclear, N/A=not applicable | | | | | | | | | |

1. Is it clear what is the cause and what is the effect?

2. Were the participants included in any comparisons similar?

3. Were the participants included in any comparisons receiving similar treatment/care, other than the exposure or intervention of interest?

4. Was there a control group?

5. Were there multiple measurements of the outcome both pre and post the intervention/ exposure?

6. Was follow up complete and if not, were differences between groups in terms of their follow up adequately described and analysed?

7. Were the outcomes of participants included in any comparisons measured in the same way?

8. Were outcomes measured in a reliable way?

9. Was appropriate statistical analysis used?

**3. Outcomes matrix of included studies as reported in the RR.**

| Authors in **Bold** denote cancer diagnostic centres | ***Arnaout et al 2013*** | *Bosch et al 2011* | *Bosch et al 2012a* | ***Bosch et al 2012b*** | *Bosch et al 2012c* | *Bosch, Jordán and López-Soto 2013* | ***Bosch et al 2018*** | ***Bosch et al 2020*** | *Bosch et al 2021* | *Brito‐Zerón, et al 2014* | ***Choudhury et al 2013*** | ***Harcourt et al 1998*** | ***McKevitt et al 2017*** | *Montori-Palacín et al 2017* | ***Nixon et al 2020*** | *Pallan et al 2005* | *Porter et al 2003* | *Sanclemente-Ansó 2016* | ***Sethukavalan et al 2013*** | ***Sewell et al 2020*** | ***Total no of studies (n=20)*** |
| --- | --- | --- | --- | --- | --- | --- | --- | --- | --- | --- | --- | --- | --- | --- | --- | --- | --- | --- | --- | --- | --- |
| Performance outcomes | | | | | | | | | | | | | | | | | | | | | |
| Wait time from confirmation of BI-RADS 5 status of abnormal diagnostic mammogram to biopsy | X |  |  |  |  |  |  |  |  |  |  |  |  |  |  |  |  |  |  |  | 1 |
| Time from biopsy to pathology verification | X |  |  |  |  |  |  |  |  |  |  |  |  |  |  |  |  |  |  |  | 1 |
| Time to first visit |  | X | X | X | X | X | X | X |  |  | X |  |  | X |  |  | X |  |  |  | 10 |
| Number of visits required to receive diagnosis |  |  |  |  |  | X |  |  | X |  |  |  |  | X |  |  | X |  |  |  | 4 |
| Number of biopsies to arrive at diagnosis |  |  |  |  |  |  |  |  |  |  |  |  |  |  | X |  |  |  |  |  | 1 |
| Time to diagnostic examination |  |  |  |  | X |  | X |  |  |  |  |  |  | X |  | X |  |  |  |  | 4 |
| Time to surgical consultation/ assessment | X |  |  |  |  |  |  |  |  |  |  |  | X |  |  |  |  |  |  |  | 2 |
| Time from consultation to treatment | X |  |  |  |  |  |  |  |  |  | X |  | X |  | X |  |  |  | X |  | 5 |
| Time from cancer suspicion to treatment |  |  |  |  |  |  |  |  |  |  |  |  |  |  |  |  |  |  | X |  | 1 |
| Time to diagnosis |  | X | X | X | X | X | X | X | X | X |  |  |  | X | X |  |  | X | X | X | 14 |
| Time from diagnosis to treatment |  |  |  |  |  |  |  |  |  |  |  |  |  |  |  |  |  |  | X |  | 1 |
| Time from diagnosis to specialist consultation |  |  |  |  |  |  |  |  |  |  |  |  |  |  |  |  |  |  | X |  | 1 |
| Referral patterns over time |  |  |  |  |  | X |  |  |  |  |  |  |  |  |  |  |  |  |  |  | 1 |
| Onward referral |  | X | X | X | X | X | X |  |  | X | X |  |  | X |  |  |  |  |  |  | 9 |
| Economic outcomes | | | | | | | | | | | | | | | | | | | | | |
| Mean cost per hospital stay |  | X | X | X | X |  | X | X |  | X |  |  |  |  |  |  | X | X |  |  | 9 |
| Mean cost per visit to the diagnostic centre |  | X | X | X | X | X | X | X | X | X |  |  |  |  |  |  | X |  |  |  | 10 |
| Average cost per process (from admission to discharge) |  | X | X | X | X | X |  |  |  |  |  |  |  |  |  |  |  |  |  |  | 5 |
| Total cost per patient |  |  |  |  |  |  | X | X | X | X |  |  |  |  |  |  |  | X |  | X | 6 |
| Cost of diagnostic tests per patient |  |  |  |  |  |  | X | X |  |  |  |  |  |  |  | X |  |  |  |  | 3 |
| Cost saving related to diagnostic investigation |  |  |  |  |  |  |  |  |  |  |  |  |  |  |  |  |  | X |  |  | 1 |
| Cost saving from hospitalisation |  |  |  |  |  |  | X |  |  |  |  |  |  |  |  |  |  | X |  |  | 2 |
| Cost saving per patient related to structural and general functioning costs of hospitalisation |  |  |  |  |  |  |  |  |  |  |  |  |  |  |  |  |  | X |  |  | 1 |
| Mean cost saving per patient |  |  |  |  |  |  |  |  |  |  |  |  |  |  |  |  |  | X |  |  | 1 |
| Overall cost saving |  |  |  |  |  |  |  |  |  |  |  |  |  |  |  |  |  | X |  |  | 1 |
| Staffing costs |  |  |  |  |  |  | X | X | X |  |  |  |  |  |  |  |  | X |  |  | 4 |
| Costs of catering/ cleaning/laundry/ travel/maintenance/ administrative/depreciation/consultation |  |  |  |  |  |  |  | X |  |  |  |  |  |  |  |  |  |  |  |  | 1 |
| Cost-effectiveness |  |  |  |  |  |  |  |  |  |  |  | X |  |  |  |  |  |  |  | X | 2 |
| Direct, Indirect and structural costs |  |  |  |  |  |  |  | X | X | X |  |  |  |  |  |  |  | X |  |  | 4 |

**4. Characteristics of included diagnostic centres**

| **Reference** | **Location & setting** | **Aim of the centre** | **Staff & facilities** | **Investigative procedures/services conducted** | **Diagnosis of interest** | **Referral** | **Referral criteria** | **Key underpinning elements of diagnostic centre** |
| --- | --- | --- | --- | --- | --- | --- | --- | --- |
| Canada | | | | | | | | |
| *Lymphoma Rapid Diagnosis Clinic (LRDC) at Princess Margaret Cancer Centre, Toronto, Ontario* | | | | | | | | |
| Nixon et al (2020). | Lymphoma **Rapid diagnosis** clinic (LRDC) **based in a tertiary cancer centre** (Princess Margaret Cancer Centre), part of University Health Network (UHN), Toronto, Ontario, Canada. | The goal of the centre was to provide **specialised comprehensive assessment and timely and appropriate investigations** to reduce wait times to a **definitive biopsy** to establish histologic diagnosis and initiation of treatment. | The clinic was **led by a Nurse practitioner;** however, it appears weekly dedicated operating rooms for lymphoma biopsies were used by head and neck, thoracic, and general surgeons, and biopsy material from outside facilities were reviewed by hemapathologists when available. | Laboratory tests, peripheral blood flow cytometry, tuberculosis skin testing, abdominal ultrasound, computed tomography scans, bone marrow biopsy, or FNA. Referral to surgical services for consideration of excisional lymph node biopsy or radiology for image guided core biopsy was requested based on location and size of lymphadenopathy. | Lymphadenopathy and suspected lymphoma | Primary care or medicine, ER, or community specialists | Lymphadenopathy on the basis of **clinical assessment or imaging, biopsy results suspicious** for lymphoma, or peripheral blood abnormalities.  Patient symptoms included: symptoms of viral infection, “B” symptoms, new pain, pruritus, palpable lymph nodes, lymphocytosis (ALC > 4.0 X 10^9^/L) | The clinic was Nurse practitioner-led  The optimal approach to lymphoma diagnosis is multidisciplinary, and our data suggest that even a modest additional investment in personnel and resources would be expected to significantly improve delivery of care for patients with suspected lymphoma. |
| *The Gale and Graham Wright Prostate Centre, North York General Hospital Toronto, Ontario* | | | | | | | | |
| Sethukavalan et al (2013). | **Rapid diagnostic** unit (RDU) called The Gale  and Graham Wright Prostate Centre, set up at the North  York General Hospital (NYGH), Branson Site, Toronto, Ontario, Canada. | The Wright Prostate Centre is a diagnostic assessment programme and **multidisciplinary clinic where both radiation oncology and urology specialists see every patient on the same day** after obtaining their **biopsy results** | Staff include **radiation oncology and urology specialists** | Unclear | Prostate cancer | Urologists or family physicians (primary care) | Patients with **suspicion of cancer** either (based on a **prostate-specific antigen (PSA) test**), because of **symptoms**, or an abnormal **digital rectal exam** (DRE), which warranted further testing or referral to a specialist to definitively diagnose or rule out cancer |  |
| *Rapid Access Diagnostic and Support (RADS) at The Women’s Breast Health Center, Ottawa Hospital, Ottawa, Ontario* | | | | | | | | |
| Arnaout et al (2013). | The Women’s Breast Health Center of the Ottawa Hospital in Ottawa, Ontario, Canada (a university-affiliated tertiary care centre) | The **Rapid Diagnosis** and Support (RADS) Program initiative was a pilot programme which aimed to accommodate the **increasing patient volume, reduce wait times, decrease fragmentation of care**, and enhance a patient’s overall experience. | A **multidisciplinary team** of breast cancer specialists (five radiologists, five surgeons, two pathologists, one nurse manager, two nurse navigators, and a diagnostic imaging manager) | Routine screening mammography, initial diagnostic imaging workup (mammogram and/or breast ultrasound) for a breast problem (e.g., palpable mass, breast pain, nipple discharge), or additional diagnostic imaging and biopsy workup following an abnormal mammogram performed at another institution | Breast cancer | Primary care | Patients are routinely referred to the breast centre for either:  1. Routine **screening** mammogram  2. **Breast symptoms** requiring diagnostic imaging workup  3. **Abnormal outside imaging** needing additional  diagnostic workup/biopsy | Coordination of diagnostic imaging workup and nursing support were provided by **a nurse navigator**. The nurse navigator was assigned to track all patients within the programme to help expedite and inform them of additional diagnostic imaging workup appointments, provide psychosocial support to the patients during their diagnostic care, and provide a telephone hotline service for which patients can call if they had any questions or concerns.  RADS program patients were labelled as such on imaging and pathology requisitions to help avoid delays. Interventions, such as coordinating same day tests and changing biopsy schedules and calling pathologists to accommodate faster access to pathology, were implemented as much as possible.  Through this prospective pilot project, we have learned that we can efficiently triage patients at highest risk of having breast cancer and therefore initiate strategies to reduce diagnostic wait times for these patients and prevent fragmentation of care. Most of these interventions were aimed **at tracking the diagnostic journey of these patients so that their workup can be appropriately expedited and unnecessary gaps in scheduling can be avoided**. |
| *Rapid Access Breast Clinic (RABC) at Mount St Joseph Hospital, Vancouver* | | | | | | | | |
| McKevitt et al (2017). | **Rapid Access** Breast Clinic (RABC) at Mt St Joseph Hospital, British Columbia, Canada | The RABC was established **to offer a single site for coordinated clinical and radiological assessment** of breast problems | Clerical staff, radiologists, surgeons, nurse navigator and clinic family physician (FP) | Offers on-site mammography, breast ultrasound, ultrasound-guided biopsy, and mammographic and ultrasound-guided fine wire localisation. Patients requiring **stereotactic core biopsy, MRI or MRI-guided biopsy have those investigations coordinated by the RABC at regional imaging sites** offering those investigations. | Breast cancer | Family physician (primary care) | Abnormal **screening** mammogram or presenting with a **new breast problem.** | The Rapid Access Breast Clinic (RABC) was established following the guidelines for breast  centres outlined by the European Society of Mastology (EUSOMA).  The clinic **provided triple evaluation** of patients with close collaboration between clinicians and radiologists, facilitated by clinical pathways and nurse navigation. The development of the RABC in conjunction with the radiology department at the centre created a unique situation in which the breast surgeons saw patients managed by two separate diagnostic pathways. |
| Spain | | | | | | | | |
| *Quick Diagnosis Unit (QDU), Hospital Clínic, Barcelona* | | | | | | | | |
| Bosch et al (2020).  Brito‐Zerón et al (2014).  Bosch, Jordán and López-Soto (2013).  Bosch et al (2012a).  Bosch et al (2012b).  Bosch et al (2012c).  Bosch et al (2011). | **Quick Diagnosis** Unit (QDU) based in the Adult Day Care Centre which is situated within the General Internal Medicine Department of a public tertiary university hospital (Hospital Clínic), Barcelona, Spain. | The QDUs implemented in  Spain are mainly **led by general internists** and  aim to provide patients with serious disease with the **prompt**  **and effective diagnosis and treatment** they deserve and  require | Staff at QDU includes a full-time consultant internist, a senior internal medicine resident, a full-time nurse, a part-time nurse coordinator, and 2 part-time administrative assistants. The unit is open 5hours a day, 5 days a week. In addition, consultants from other services are provided as required.  It has a consulting room and a waiting room for patients and families, and functions daily. | Upper gastrointestinal endoscopic ultrasound (EUS), US/CT-guided biopsy, contrast-enhanced thin-slice CT scan of the chest, abdomen, and pelvis  Laboratory tests included, among others, acute phase reactants (C-reactive protein, erythrosedimentation rate), hemogram (total leucocytes, manual white blood cell count, haemoglobin, haematocrit, platelets), liver function tests, serum lactate dehydrogenase, serum total proteins and protein electrophoresis, microbiological serologies [e.g. IgM and IgG for cytomegalovirus (CMV), Epstein–Barr virus (EBV), Toxoplasma gondii, human parvovirus B19], HIV testing, b2 microglobulin, specific serum tumour markers, specific serum autoantibodies, specific genetic studies (autoinflammatory diseases), including cultures, imaging studies, endoscopies and cytology/biopsy studies  The main diagnostic tests (analytical and microbiological tests, simple radiology [X-ray], computed tomography [CT], echography, nuclear scintigraphy, digestive endoscopy, biopsies and lymph node fine-needle puncture aspiration [FNPA]) are normally performed within 7 days after the first visit. | Most studies were non-specific but including potentially severe diseases such as **cancer and severe anaemia**  (some studies had a specific focus including severe anaemia, pancreatic cancer, unexplained fever). | Primary care and emergency department | The QDU evaluates stable **patients with** **suspected serious disease who require expeditious workup** and who, in many instances, would be admitted to hospital for diagnostic testing. Thus, patients must be physically and mentally capable of  attending several outpatient appointments.  Symptoms may include: Anaemia, cachexia-anorexia syndrome, febrile syndrome, adenopathies and/or palpable masses, unexplained severe abdominal pain, chronic diarrhoea, recent severe constipation, rectorrhagia, jaundice, lung and/or pleural abnormalities, unexplained dyspnea, dysphagia, ascites, anasarca, bone pain with suspicion of malignancy, arthritis, hemogram abnormalities suggestive of primary hematologic disorder, splenomegaly and/or hepatomegaly, monoclonal paraprotein band with or without suspicion of multiple myeloma, neurologic disorders (central, spinal, and peripheral nervous system) | The operation of the QDU is based on an urgent first visit, followed by the preferential scheduling and coordination of complementary tests and subsequent visits until a diagnosis is made. |
| *Quick Diagnostic Unit (QDU) at Bellvitge Hospital, Barcelona* | | | | | | | | |
| Sanclemente-Ansó et al (2016). | QDU at Bellvitge University Hospital in Barcelona, which is affiliated to the University of Barcelona, Catalonia, Spain. The QDU is integrated to the Internal Medicine Department of the hospital. | Not stated | The QDU is **internist led** and open 7 hours a day, 2 days a week (Tuesdays and Fridays). Analysis implies staff also included attending physician, registered nurse, caretaker. | Not explicitly stated, but to include blood and urine analysis, X-ray CT, simple X-ray, PET-CT, biopsy, bronchoscopy, cytology, microbial culture, scintigraphy, mammography, specialist consultation, ultrasonography, colonoscopy, electrocardiography, lower and upper gastrointestinal series, esophagogastroduodenoscopy, blood marrow aspiration, flow cytometry | Non-specific but including **potentially serious diseases** | Primary care and the emergency department | Anaemia, cachexia-anorexia syndrome, febrile syndrome, adenopathies and/or palpable masses, unexplained severe abdominal pain, chronic diarrhoea , recent severe constipation, rectorrhagia, jaundice, lung and/or pleural abnormalities, unexplained dyspnea, dysphagia, ascites, anasarca, bone pain with suspicion of malignancy, arthritis, hemogram abnormalities suggestive of primary hematologic disorder, splenomegaly and/or hepatomegaly, monoclonal paraprotein band with or without suspicion of multiple myeloma, neurologic disorders (central, spinal, and peripheral nervous system) | Hospital-based QDUs are normally led by internists and are a **distinct model of outpatient care delivery** almost exclusively reported in Spain, most notably in Catalonia. The driving reason explaining the important role of internists leading QDUs is the common presence of **nonspecific symptoms such as weight loss, fatigue, malaise or fever of unknown origin in patients referred to these units**. The **versatility of these physicians**  **for the diagnosis of a wide range of serious disorders together with their integral, global view of the patient** contrast with the more specialized approach of physicians at other units such as the UK one-stop diagnostic clinics.  The requirements for evaluation by the QDU at Bellvitge Hospital are: 1) **clear referral criteria based on a pre-established list of suspected serious disorders**; 2) the first visit has to occur as soon as possible after referral (≤15 days); 3) patients must have preferential access to a wide range of diagnostic tests; and 4) patients should be able to attend several appointments for outpatient visits and diagnostic tests. |
| *Quick Diagnosis Unit (QDU), Hospital Plató* | | | | | | | | |
| Bosch et al (2021).  Montori-Palacín et al (2017). | QDU of a second-level district hospital (Hospital Plató) with 160 beds and providing healthcare for a population of 140,000 inhabitants. | The unit works as an **ambulatory clinic** evaluating patients with **suspected severe conditions** whose physical performance allows them to travel from home to hospital and back for visits and examinations. | The unit is staffed with two part-time **general internists** each working 4 hours per week, as well as administrative personnel. | Not described but appear to include CT scan, MRI, ultrasonography, endoscopy, scintigraphy, body FDG-PET, cytology/biopsy, bone marrow aspiration. | **Suspected severe conditions** | Primary care, emergency department, outpatient clinics, and inpatient wards. | Not described in detail, but included unintentional weight loss, adenopathies and/or palpable mases, anaemia, fever, gastrointestinal symptoms, test abnormalities, osteoarticular symptoms, respiratory symptoms, unexplained tiredness, neurological disorders, ascites and other reasons (not defined). | The general working protocol of the unit consists of a **rapid first appointment after referral from primary care** centres or the emergency departments of the hospitals  (usually within 5 days), followed by **preferential programming of diagnostics tests and subsequent visits until a diagnosis** is made. |
| United Kingdom | | | | | | | | |
| *The Breast Care Centre, Bristol* | | | | | | | | |
| Harcourt et al (1998). | **One stop clinic** in a hospital (location not given but likely to be study author’s centre - The Breast Care Centre, Frenchay Healthcare Trust, Bristol, UK) | Not stated | All clinics were conducted by the same **two surgeons** (one consultant, one staff grade) | **Triple assessment** (consisting of clinical examination, ultrasound scanning and cytology) and mammography, when needed. | Breast cancer | GP (primary care) | Women with no previous diagnosis of breast cancer, living within reasonable travelling distance of the hospital and whose general practitioner (GP) referral letter stated the presence of a breast lump | Triple assessment of each woman  was conducted by either member of staff in  the initial appointment. Mammography, when needed, was then carried out by radiologists in the hospital’s general X-ray department. Cytology specimens were analysed in the  pathology department sited elsewhere in the hospital. This system was constant over the course of the study. |
| *Rapid Diagnostic Clinic (RDC) at St Barts Health NHS Trust, London* | | | | | | | | |
| Choudhury et al (2013). | A ‘**Rapid Diagnostic** Clinic’ (RDC) within the ENT department (of Barts Health NHS Trust, London) | The RDC provides **multi-modality specialist assessment** for new referrals, with on-site sonography and cytology. | ENT head and neck specialist, either a consultant or specialist registrar, consultant head and neck radiologist, consultant histopathologist.  This clinic is run on a weekly basis. | Ultrasonography, and fine needle aspiration cytology (FNAC) | Head and neck cancer | GPs and other specialities within the hospital | Not clearly stated. (Targeted referrals included patients revered via the 2WW scheme and non-2WW referrals) | The RDC in this study was established based on National Institute of Clinical Excellence (NICE) recommendations for improving healthcare services for head and neck cancers. The RDC is a **one-stop diagnostic service** where all new target referrals for patients with suspected malignancy can receive **multi-modality specialist assessment**. |
| *Demyelinating disease diagnostic clinic (DDC) at University College London* | | | | | | | | |
| Porter et al (2003). | Demyelinating disease diagnostic clinic (DDC) at the National Hospital for Neurology and Neurosurgery, University College London  Hospitals | The DDC aims to **minimize the time between referral and completion of tests** by carrying out tests on the **same day** and providing **follow-up within four weeks.** | The clinic is staffed by a consultant neurologist and a multiple sclerosis nurse specialist | **Evoked potential (VEP) testing, magnetic resonance imaging (MRI), and blood screening** | **Multiple sclerosis** | GP or another neurologist | GPs tend to refer patients with new **neurological symptoms**, lasting days or weeks with **no obvious explanation**. Neurologists refer **difficult diagnostic cases** including suspected primary progressive disease, those with an atypical presentation and those with nonorganic symptoms | The DDC was modelled on the UK multiple sclerosis Society standards of healthcare set for the diagnostic phase.  The structure of the DDC provides an **appropriate setting and experienced professional support to deal with the initial**  **psychological impact of the diagnosis** and offers ongoing support through patient/family health promotion and self-management programmes.  The clinic is designed to allow a 45-minute new patient appointment with the consultant neurologist and MS nurse, followed by access to visual evoked potential (VEP) testing, magnetic resonance imaging (MRI) and blood screening, as appropriate. Follow-up appointments to all patients are scheduled to allow 30 minutes with the neurologist and MS nurse followed by a further 30 minutes with the MS nurse in a quiet room. All **newly diagnosed are followed up with one to one appointments with their families** and have access to a structured educational programme entitled ’Working together to understand MS’. |
| *Rapid Diagnostic Centre (RDC) at Neath Port Talbot Hospital, Neath, Wales* | | | | | | | | |
| Sewell et al (2020). | Rapid diagnostic centre (RDC), Neath Port Talbot Hospital, Wales, UK | Not stated | Consultant physician, a radiologist, a clinical nurse specialist (CNS), and a healthcare support worker (HCSW). Management and clinical guidance are provided by the RDC coordination manager and GP project lead. Two half-day clinics a week with five available clinic slots | It is unclear if the full range of investigative procedures available at the centre was reported but includes physical examination and computed tomography (CT) | Cancer | GP (primary care) | Patients with **vague and/or non-specific symptoms suspicious of cancer, who do not meet criteria for referral under an urgent suspected cancer (USC) pathway** | This pilot rapid diagnosis centre (RDC) allows  GPs within targeted clusters to refer adults  with vague and/or non-specific symptoms  suspicious of cancer, who do not meet criteria  for referral under an urgent suspected cancer  (USC) pathway, to a multidisciplinary RDC clinic where they are seen within a week. Patients leave the clinic with either a diagnosis and management plan or further investigations booked. |
| *Community Diagnostic Service throughout the West Midlands* | | | | | | | | |
| Pallan et al (2005). | Community based, **mobile diagnostic ultrasound service,** in a primary care area in the West Midlands, England, UK | Not stated | The service is staffed by an **independent radiographer** | Ultrasound scans included abdominal, pelvic, transvaginal, renal, and prostate | Not specified | GP (primary care) | Not stated | The mobile diagnostic ultrasound service was radiographer-led and was provided to GPs by an independent radiographer. |

**5. Potential overlap of data for the studies conducted in Spain**

| **Spanish Studies:**  Includes 11 individual studies investigating four diagnostic centres  NB: Groupings are by hospital site | | | | | |
| --- | --- | --- | --- | --- | --- |
| **Reference** | **Study duration** | **Population** | **Outcomes** | **Study comparison details** | **Information on potential data crossover** |
| ***Hospital Clínic de Barcelona*** *– Barcelona - described as an 885 bed tertiary hospital with a reference population of around 550,000. Earlier sources describe it as a 840 bed tertiary hospital with a reference population of around 540,000* | | | | | |
| Bosch et al (2021). [A comparative cost analysis between two quick diagnosis units of different levels of complexity](https://pubmed.ncbi.nlm.nih.gov/33709770/). Journal of Comparative Effectiveness Research, 10(5), pp.381-392. | 2009 to 2017 | Aged ≥18 years attending the two QDUs between January 2009 and January 2017  Sample size: 407 patients from each unit (the tertiary unit sample were randomly selected from 6,960 consecutive patients) | Participant characteristics  Referral source  Referral reason  Number of visits  Time to diagnosis  Diagnosis  Mean cost per visit  Mean cost per patient  Direct and structural costs  Indirect costs  Personnel costs  Diagnostic investigation costs | QDU of Hospital Clínic (tertiary unit) vs QDU of **Hospital Plató** (secondary unit) | Unclear |
| Bosch et al (2020). [What is the relevance of an ambulatory quick diagnosis unit or inpatient admission for the diagnosis of pancreatic cancer? A retrospective study of 1004 patients.](https://www.ncbi.nlm.nih.gov/pmc/articles/PMC7440208/pdf/medi-99-e19009.pdf)  Medicine, 2020, vol. 99, num. 11, p. e19009. | 2005 to 2018 | Aged ≥ 18 years with diagnosis of pancreatic adenocarcinoma referred to both settings  Sample size: 508 patients from QDU and 496 inpatients | Participant characteristics  Referral source  Time to first visit  Time to diagnosis  Risk factors for cancer  Predictors of hospitalisation  Mean tumour size  Tumour site  Cancer stage on presentation  Charlson comorbidity index  Surgical characteristics  Mean cost per hospital stay  Mean cost per visit  Mean cost per patient  Cost of diagnostic tests  Personnel costs  Costs of catering/cleaning/laundry/travel/  maintenance/administrative/depreciation/  consultation | QDU of Hospital Clínic vs inpatient setting at same hospital | Potential QDU data overlap with Bosch 2021 (i.e pancreatic ca data only between 2009 and 2017) |
| Montori-Palacín et al (2017). [Quick outpatient diagnosis in small district or general tertiary hospitals: A comparative observational study](https://www.ncbi.nlm.nih.gov/pmc/articles/PMC5459703/). Medicine 96 (22). | 2009 to 2016 | Aged ≥18 referred to both settings (chosen randomly)  Sample size: 866 participants, 336 from QDU1 and 530 from QDU2 | Participant characteristics  Referral source  Referral reason  Time to first visit  Number of visits  Time to examination  Time to diagnosis  Diagnosis  Diagnostic tests  Onward referrals | QDU1 (**Hospital Plató**) vs QDU2 (Hospital Clínic) | Potential QDU1 and 2 data overlap with Bosch 2021 (i.e patient data between 2009 and 2016) – note that cases were chosen randomly |
| Bosch et al (2018). [Time to diagnosis and associated costs of an outpatient vs inpatient setting in the diagnosis of lymphoma: a retrospective study of a large cohort of major lymphoma subtypes in Spain](https://www.ncbi.nlm.nih.gov/pmc/articles/PMC5848556/pdf/12885_2018_Article_4187.pdf) BMC cancer, 18(1), pp.1-15. | 2006 to 2016 | Aged ≥18 years with lymphoma (4 types)  Sample size: 1,779 patients. 1,184 outpatients (688 from QDU1 and 496 from QDU 2) and 535 inpatients | Patient characteristics  Referral source  Time to first visit  Number of visits  Time to examination  Time to diagnosis  Diagnostic tests  Diagnosis  Onward referral  Mean cost per hospital stay  Mean cost per visit  Mean cost per patient  Cost of diagnostic tests  Cost saving from hospitalisation  Personnel costs | QDU1 (Hospital Clínic) vs inpatient wards (within Hospital Clínic) vs QDU2 of **hospital of Bellvitge** | Potential QDU1 data overlap with Bosch 2021 (i.e lymphoma data only between 2009 and 2016) |
| Brito‐Zerón et al. (2014). [Diagnosing unexplained fever: can quick diagnosis units replace inpatient hospitalization?](https://onlinelibrary.wiley.com/doi/pdf/10.1111/eci.12287) European Journal of Clinical Investigation, 44(8), pp.707-718. | 2008 to 2011 | All consecutive patients referred to the QDU between November 2008 and April 2011 due to undiagnosed fever  Sample size: 344 patients, 176 from QDU and 168 controls (internal medicine department) | Participant characteristics  Referral source  Referral reason  Number of visits  Time to diagnosis  Diagnosis  Length of hospital stay  Diagnostic tests  Death during evaluation  Onward referrals  Mean cost per hospital stay  Mean cost per visit  Mean cost per patient | QDU (Hospital Clínic) vs internal medicine department (unclear if same wards as in Bosch 2020 and Bosch 2018) | Potential QDU data overlap with Bosch 2021, i.e FUN data only between 2009 and 2011 |
| Bosch, Jordán and López-Soto (2013). [Quick diagnosis units: avoiding referrals from primary care to the ED and hospitalizations](https://www.clinicalkey.com/service/content/pdf/watermarked/1-s2.0-S0735675712003385.pdf?locale=en_US&searchIndex=) The American Journal of Emergency Medicine, 31(1), pp.114-123 | 2006 to 2012 | Consecutive patients attending the QDU and patients admitted to the internal medicine service (randomly chosen) between September 2006 and January 2012  Sample size: 4,170 QDU patients and 3.030 hospitalised patients | Participant characteristics  Referral source  Referral reason  Referral appropriateness  Time to first visit  Number of visits  Referral patterns over time  Time to diagnosis  Diagnosis  Length of hospital stay  Death during evaluation  Onward referrals  Mean cost per hospital stay  Mean cost per process  Mean cost per visit  Patient preferences | QDU patients (Hospital Clínic) vs patients admitted to the internal medicine service | QDU data overlap with Bosch 2012a (2,000 consecutive patients evaluated between 2007 and 2010**)** |
| Bosch et al (2012a). [Quick diagnosis units versus hospitalization for the diagnosis of potentially severe diseases in Spain](https://shmpublications.onlinelibrary.wiley.com/doi/abs/10.1002/jhm.931) Journal of Hospital Medicine, 7(1), pp.41-47 | 2006 to 2010 | Consecutive QDU patients evaluated between December 2007 and July 2010 and patients diagnosed with anaemia (n = 548), cachexia-anorexia syndrome (n = 458), febrile syndrome (n = 240), and adenopathies or palpable masses (n = 208) admitted to the internal medicine department between September 2006 and June 2010.  Sample size: 2,000 QDU patients and 1,454 patients admitted to the internal medicine department | Participant characteristics  Referral source  Referral reason  Time to first visit  Number of visits  Time to diagnosis  Diagnosis  Length of hospital stay  Charlson comorbidity index  Hospital bed days saved  Onward referrals  Cost per hospital stay  Cost per process  Cost per visit  Patient satisfaction  Patient preferences | QDU patients vs patients admitted to the internal medicine department | QDU data overlap with Bosch, Jordán and López-Soto 2013 (2,000 consecutive patients evaluated between 2007 and 2010) |
| Bosch et al (2012b). [Comparison of Quick Diagnosis Units and Conventional Hospitalization for the Diagnosis of Cancer in Spain: A Descriptive Cohort Study](https://www.karger.com/Article/Abstract/341658) Oncology, 83(5), pp.283-291. | 2008 to 2010 | QDU patients with both initially suspected and ultimately confirmed (pathologically proven) cancer evaluated between November 2008 and April 2010**.** Also included patients newly diagnosed with cancer admitted to the internal medicine department  Sample size: 169 (62.8% of 269 consecutive patients evaluated for inclusion) QDU patients, and 53 Hospitalised patients | Patient characteristics  Referral source  Reason for referral/consultation  Time to first visit  Number of visits  Time to diagnosis  Length of hospital stay  Diagnosis  Onward referral  Mean cost per hospital stay  Mean cost per visit  Mean cost per process | QDU (Hospital Clínic) vs internal medicine department | potential QDU data overlap with above studies by Bosch et al. |
| Bosch et al (2012c). [Quick diagnosis units or conventional hospitalisation for the diagnostic evaluation of severe anaemia: A paradigm shift in public health systems?](https://www.clinicalkey.com/service/content/pdf/watermarked/1-s2.0-S0953620511000458.pdf?locale=en_US&searchIndex=) European Journal of Internal Medicine, 23(2), pp.159-164. | 2006 to 2010 | Consecutive patients with severe anaemia attended by the QDU and consecutive patients with the same diagnosis admitted to the internal medicine department  Sample size: 282 cases from QDU, 252 hospitalised patients | Participant characteristics  Referral source  Referral reason  Time to first visit  Number of visits  Time to examination  Time to diagnosis  Diagnosis  Length of hospital stay  Charlson morbidity index  Diagnostic tests  Treatment received at unit  Onward referrals  Mean cost per hospital stay  Mean cost per process  Mean cost per visit  Patient satisfaction  Patient preferences | QDU (Hospital Clínic) vs internal medicine department | Likely overlap of patient data with above studies |
| Bosch et al (2011). [Outpatient Quick Diagnosis Units for the evaluation of suspected severe diseases: an observational, descriptive study.](https://www.ncbi.nlm.nih.gov/pmc/articles/PMC3109368/pdf/cln-66-05-737.pdf)  Clinics, 66(5), pp.737-741. | 2008 to 2010 | Consecutive patients evaluated in the QDU  Sample size: 1,000 QDU patients and ?150 patients admitted to internal medicine department | Participant characteristics  Referral source  Referral reason  Referral appropriateness  Time to first visit  Number of visits  Time to diagnosis  Diagnosis  Length of hospital stay  Diagnostic tests  Hospital bed days saved  Onward referral  Mean cost per hospital stay  Mean cost per process  Mean cost per patient  Patient satisfaction  Patient preference | QDU (Hospital Clínic) vs internal medicine department | Likely overlap of patient data with above studies |
| **Bellvitge Hospital –** Barcelona **-** described as a 750 bed tertiary hospital with a reference population of around 350,000 | | | | | |
| Sanclemente-Ansó et al (2016). [Cost-minimization analysis favors outpatient quick diagnosis unit over hospitalization for the diagnosis of potentially serious diseases](https://pubmed.ncbi.nlm.nih.gov/26944565/). Eur J Intern Med; 30:11-17. doi: 10.1016/j.ejim.2015.12.015 | 2008 to 2012 | Patients diagnosed with severe anaemia, lymphoma, and lung cancer selected from consecutive patients referred to the unit between March 2008 and June 2012 and those electively hospitalised at the Internal Medicine Department of the hospital for diagnostic workup during the same period and who had the same final diagnoses.  Sample size: 195 QDU patients and 237 Internal Medicine department patients | Referral source  Number of visits  Time to diagnosis  Diagnosis  Length of hospital stay  Diagnostic test  Mean cost per hospital stay  Mean cost per patient  Direct and structural costs  Indirect costs  Personnel costs  Diagnostic investigation costs  Mean cost saving per patient  Overall cost saving  Diagnostic investigation cost saving  Hospitalisation cost saving  Cost saving per patient | QDU (Bellvitge) vs internal medicine department | Potential QDU data overlap with Bosch 2018 (QDU2) |
